# Multiparametric analysis of anti-proliferative and apoptotic effects of gold nanoprisms on mouse and human primary and transformed cells, biodistribution and toxicity in vivo

**DOI:** 10.1186/s12989-017-0222-4

**Published:** 2017-10-26

**Authors:** Marta Pérez-Hernández, María Moros, Grazyna Stepien, Pablo del Pino, Sebastián Menao, Marcelo de las Heras, Maykel Arias, Scott G. Mitchell, Beatriz Pelaz, Eva M. Gálvez, Jesús M. de la Fuente, Julián Pardo

**Affiliations:** 1 Departamento de Bioquímica y Biología Molecular y Celular, Facultad de Ciencias, Universidad de Zaragoza, 50009 Zaragoza, Spain; 2 Instituto de Investigación Sanitaria de Aragón (IIS Aragón), Centro de Investigación Biomédica de Aragón (CIBA), Universidad de Zaragoza, 50009 Zaragoza, Spain; 3 Instituto Universitario de Nanociencia de Aragón (INA), Universidad de Zaragoza, 50018 Zaragoza, Spain; 4 Institute of Applied Sciences and Intelligent Systems-CNR, Via Campi Flegrei, 34, 80078 Pozzuoli, Italy; 5 Fundación Instituto Universitario de Nanociencia de Aragón (FINA), Universidad de Zaragoza, 50018 Zaragoza, Spain; 6 CIBER in Bioengineering, Biomaterials and Nanomedicine (CIBER-BBN), Zaragoza, Spain; 7 Centro Singular de Investigación en Química Biológica y Materiales Moleculares (CiQUS) y Departamento de Física de Partículas, Universidade de Santiago de Compostela, 15782 Santiago de Compostela, Spain; 8 Departamento de Bioquímica clínica. H.C.U. Lozano Blesa, 50009 Zaragoza, Spain; 9 Departamento de Patología Animal, Facultad de Veterinaria, Universidad de Zaragoza, 50009 Zaragoza, Spain; 10 Instituto de Ciencia de Materiales de Aragón, CSIC-Universidad de Zaragoza, Zaragoza, Spain; 11 Instituto de Carboquímica ICB-CSIC, 50018 Zaragoza, Spain; 12 Departamento de Microbiología, Medicina Preventiva y Salud Pública, Facultad de Medicina, Universidad de Zaragoza, 50009 Zaragoza, Spain; 13 Aragón I+D Foundation (ARAID), Gobierno de Aragón, Zaragoza, Spain

**Keywords:** Gold nanoprisms, Nanotoxicology, ROS generation, Mitochondrial membrane-potential, Biodistribution, In vivo

## Abstract

**Background:**

The special physicochemical properties of gold nanoprisms make them very useful for biomedical applications including biosensing and cancer therapy. However, it is not clear how gold nanoprisms may affect cellular physiology including viability and other critical functions. We report a multiparametric investigation on the impact of gold-nanoprisms on mice and human, transformed and primary cells as well as tissue distribution and toxicity in vivo after parental injection.

**Methods:**

Cellular uptake of the gold-nanoprisms (NPRs) and the most crucial parameters of cell fitness such as generation of reactive oxygen species (ROS), mitochondria membrane potential, cell morphology and apoptosis were systematically assayed in cells. Organ distribution and toxicity including inflammatory response were analysed in vivo in mice at 3 days or 4 months after parental administration.

**Results:**

Internalized gold-nanoprisms have a significant impact in cell morphology, mitochondrial function and ROS production, which however do not affect the potential of cells to proliferate and form colonies. In vivo NPRs were only detected in spleen and liver at 3 days and 4 months after administration, which correlated with some changes in tissue architecture. However, the main serum biochemical markers of organ damage and inflammation (TNFα and IFNγ) remained unaltered even after 4 months. In addition, animals did not show any macroscopic sign of toxicity and remained healthy during all the study period.

**Conclusion:**

Our data indicate that these gold-nanoprisms are neither cytotoxic nor cytostatic in transformed and primary cells, and suggest that extensive parameters should be analysed in different cell types to draw useful conclusions on nanomaterials safety. Moreover, although there is a tendency for the NPRs to accumulate in liver and spleen, there is no observable negative impact on animal health.

**Electronic supplementary material:**

The online version of this article (10.1186/s12989-017-0222-4) contains supplementary material, which is available to authorized users.

## Background

The intrinsic optical properties of metal nanoparticles render them as versatile tools for a variety of bioapplications including, but not limited to, imaging, therapy and diagnosis [1–4]. In particular, anisotropic gold nanoparticles (NPs) such as nanorods [5], nanoshells [6], or nanoprisms [7, 8] show great promise for in vivo applications [5, 9–12]. Three main facts support the use of anisotropic gold NPs. First, the optical activity of these materials can be tuned along the NIR range, in the so-called biological window where absorption by physiological components is less restrictive [13]. Second, in contrast to other metal NPs made of silver or copper, gold is more noble and is considered one of the biologically safest element [11]. And finally, the abundance of synthetic approaches currently available offers researchers the possibility of engineering the size, shape and surface of these materials, enabling tailoring for specific purposes [11, 14].

In parallel to investigations concerning the development of new nanomaterials with optimal bio-performance (i.e. stability and performance for working in complex biological scenarios), testing the interactions of these materials with the living world has become a major issue for the successful development of bio-nanotechnology [12, 15]. Past, present and future data concerning the impact of nanomaterials on biological systems will greatly influence the future roadmap of nanomaterials for bioapplications [16]. Materials aimed for bioapplications should preserve their original physicochemical properties in physiological conditions, but should also avoid impairing or compromising the proper function of biological entities such as proteins and cells. Nanotoxicology is a rapidly growing area, which is devoted to investigate such nano-bio interactions [17, 18]. Typically, the physicochemical properties of NPs including composition (inorganic core and surface coating), size, shape, crystallinity, and hydrophobicity can be correlated with their toxicity in vitro, which however can also vary among distinct cell lines and/or dose [14]. Yet no matter which NP model is being assayed, there are some common adverse effects [19] which can be triggered by the intrinsic dimensions of the NP under study [20]. Upon exposure to NPs the most common cellular response involves the generation of ROS, which can be tolerated up to certain levels without side effects, typically depending on the cell type [19, 21]. In addition, alteration of the cellular homeostasis due to massive accumulation of NPs inside cells or interaction with signalling pathways can lead to a multitude of adverse effects [19, 22]. It therefore stands to reason that choosing the most appropriate components and dose might prevent to some extent an adverse impact on cells. Toxicity issues triggered by release of toxic ions from metallic NPs composed of Ag [23] and Cu, or QDs containing Cd, are well documented in the literature [14]. In contrast, gold NPs coated with densely packed thiolated chains of polyethylene glycol (PEG), as used here, are believed to be among the safest NPs [24, 25].

Regarding near infrared (NIR) -active plasmonic materials, the toxicity of gold nanorods and nanoshells has been widely studied in vitro and in vivo and photothermal therapy using these materials is already in phase 1 clinical trials for the treatment of different cancer types (see details for AuroLase® Therapy) [26]. In contrast, the impact of gold NPRs on cell cultures has yet to be investigated or characterized in detail, a prerequisite before analysing its safety and potential applications in in vivo models. The molar extinction coefficient of gold NPRs can be considerably larger to those of nanorods or nanoshells, [27] and therefore the interaction with incident light can be more efficient, leading to positive effects for various bioapplications such as photothermal therapy [7, 8], optoacoustic imaging [28], or plasmonic driven thermal biosensing [13, 29].

We have recently used different types of NPs to analyse the influence of shape on the interaction between NPs and cells and provide preliminary data on the potential toxic effects in vitro [20]. As a continuation of that work here we have focused on gold NPRs to perform a more extensive and detailed study of its effect on several common cellular parameters including ROS generation, mitochondria membrane potential, proliferation, cell morphology and apoptosis, employing a variety of cell models and two primary cell lines. Moreover, we have monitored tissue distribution and the main serum biomarkers of organ damage and inflammatory response in a mouse model in vivo in order to analyze the potential adverse effects of employing NPRs as carriers in theragnostic applications.

## Results

### Characterization of NPRs

NPRs are thin (ca. 10 nm), flat single crystals, which present three congruent edge lengths of ca. 150 nm (Additional file 1: Figure S1). The surface of the NPRs was saturated with heterofunctional PEG chains (thiol-PEG-carboxy, 5 kDa). PEGylation was achieved by using heterofunctional PEG chains (HS-PEG-COOH), which bind covalently to the gold surface through its thiol group. The carboxylic end group was used for further surface functionalization with 4-aminophenyl-β-D-glucopyranoside and/or a fluorescence reporter, 5-TAMRA cadaverine (an amine-modified rhodamine) using a carbodiimide crosslinker (more details in the materials section) (Additional file 1: Figure S2 and S3). Functionalization of NPs with glucose enhances both selectivity and uptake by target cells [20]. The rhodamine dye, TAMRA, was used as a fluorescent marker to facilitate the localization of the NPRs within cells by confocal microscopy. ζ–potential measurements and the different electrophoretical mobility among the differently-modified NPRs confirmed successful NPR modifications. Additionally, fluorescence spectroscopy measurements confirmed that NPRs were susscessfully modified with TAMRA (see Additional file 1: Figure S2-S3). Finally, the colloidal stability of the NPRs was evaluated incubating a suspension of NPRs in Dulbecco’s modified Eagle’s medium (DMEM) supplemented with 10% fetal bovine serum (FBS), 1% L-glutamine, and 1% penicillin/streptomycin during 24 h at room temperature. Then, the UV-vis spectra of the NPRs was measured and compared with the spectra before incubation in cell media. This test proved that the NPRs retain their colloidal stability in the conditions used for the cell studies with no sign of aggregation (see Additional file 1: Figure S4). Four NPR models were incubated with the cells: PEGylated NPRs (NPR-P), PEGylated NPRs modified with rhodamine only (NPR-PT), glucose (NPR-PG), and both molecules (NPR-PTG).

### Internalization of NPRs by cells

We chose six common cell lines, i.e. three from human (A549: adenocarcinoma alveolar basal epithelial cells; MiaPaca: pancreatic carcinoma cells; and HeLa: cervical cancer cells) and three from mouse (MEF-SV40: embryonic fibroblasts immortalized with the virus SV40; B16: melanoma cells; and MC57G: fibrosarcoma cells). Several key factors brought us to select these cells from other common cell models. In the first instance, since we plan to test the effect of NPRs in a mouse model in vivo, the use of mouse cells in vitro is very useful to help to understand the upcoming in vivo results. Further, results in the mouse model require validation in a human system, the end potential target of this technology. In addition, since the grand aim of this research is to use NPRs as photothermal probes for cancer treatment and tumoral cell lines were chosen as the primary targets. Also, MEF-sv40 cells were chosen as a model of fibroblast non-tumoral cells since although they are immortalised, they are not tumorigeneic and thus they are usually used as a model of primary-like fibroblast cells. Lastly, as genuine primary cells, we used some of the main cell types interacting with and potentially responding against NPs after in vivo parental administration, peripheral blood mononuclear cells (PBMCs) and macrophages, the cells involved in the clearance of exogenous materials.

**Fig. 1:**
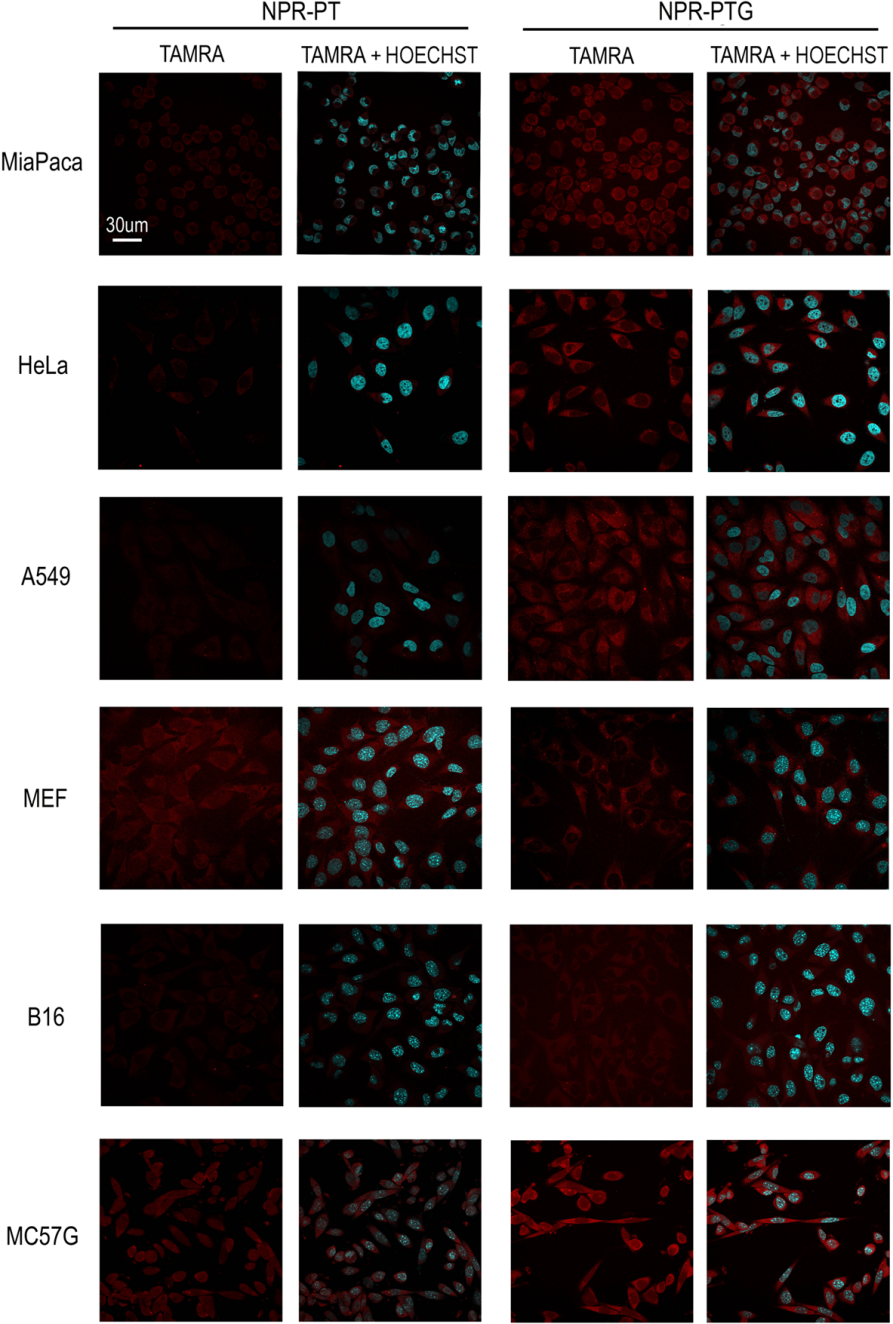
Analysis of nanoparticles internalized within cells using confocal microscopy. The indicated cells were incubated with NPR-PT and NPR-PTG nanoparticles at four different concentrations (25, 50, 100 and 200 μg/mL) for 24 h and were fixed with 4% PFA and dyed with Hoechst 3342 (DNA), pictures were taken using a confocal microscope as indicated in experimental section. A representative experiment 200 μg/mL of NPR-PT and 100 μg/mL of NPR-PTG is shown. Scale bar: 30 μm

First, the uptake of the different types of NPs was analysed in the target cells in order to ensure that NPs interact and are internalized by those cell types. Accordingly, NPRs were added to the cells at gold concentrations ranging from 25 to 200 μg/mL, commensurate with values typically used in similar studies [22]. The change in side-scattered light (SSC) was analysed by flow cytometry, which is proportional to cell morphology (granularity and intracellular complexity). As shown in Additional file 1: Figure S5 there was an increase in the SSC of the cells incubated with all types of NPRs, and this change was particularly pronounced in the case of NPRs containing glucose indicating that cells changed their intracellular morphology after interaction with NPRs, a change that could be related to NPR uptake. The results of fluorescence confocal microscopy shown in Fig. 1 at two representative concentrations (100 and 200 μg/mL), confirm that NPRs were readily internalized and accumulated inside the cells. As expected, the level of internalization varied from one cell type to another, likely due to the intrinsic endocytic properties of each cell type. Independently of the differences in the NPRs uptake between various cell types, the influence of surface coating on the internalization can be clearly observed in Fig. 1. NPR-PT probes were only poorly internalized by almost all of the cells lines, whereas coating with glucose (NPR-PTG probes) highly increased the level of internalization in all cell lines, even when the concentration of NPR-PT was doubled (200 μg/mL) in comparison with NPR-PTG. NPR-PT probes are labelled with the dye, but in terms of surface coating they are covered by a densely packed PEG coating, which, as shown in Fig. 1, prevents cell uptake, in agreement with previous results [20, 30].

### ROS generation by cells upon interaction with NPs

ROS generation was investigated in all of the cell lines after 48 h of incubation, using increasing concentrations of two NPR probes (dye labelled probes were excluded because of fluorescence interference with the test). There are several fluorogenic probes commonly employed for selective detection of ROS such as ${\mathrm{O}}_2^{-}$, mitochondrial ${\mathrm{O}}_2^{-}$, H_2_O_2_ or ONOO^−^. However, the reliability and feasibility of these tests for detection and quantification of intracellular ROS have been questioned [31, 32]. In the context of ROS generation triggered by the interaction of NPRs with cells, they are however widely employed. Here, we used dihydroethidine (2HE) for “catching” intracellular O_2_^-^, enabling for qualitative detection of the fluorescence product 2-hydroxyethidium (2-OH-E^+^). We decided to analyse the superoxide anion since this is the first and most abundant oxygen radical generated during the physiology and pathophysiology of ROS.

**Fig. 2:**
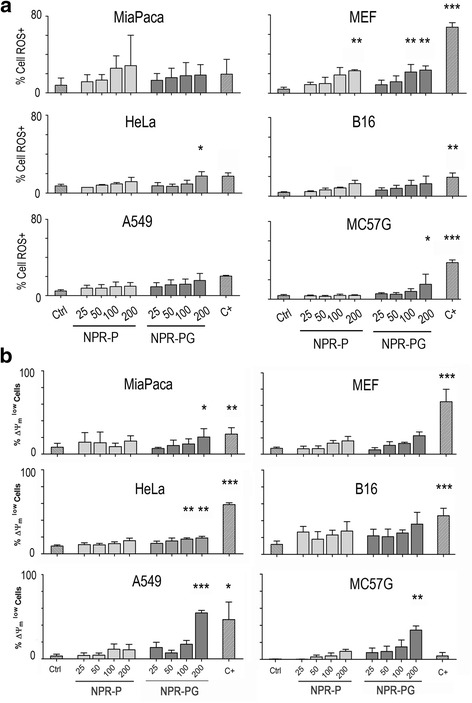
Analysis of the effect of NPRs on ROS production and loss of mitochondrial membrane potential by flow cytometry**.** Analysis of the superoxide anion generation by flow cytometry. The indicated cells were mock treated (ctrl) or incubated with NPR-P and NPR-PG nanoparticles at four different concentrations (25, 50, 100 and 200 μg/mL) for 48 h. (**a**) Superoxide generation was monitored using 2HE and (**b**) ΔΨ_m_ was monitored using DIOC_6_ as indicated in experimental section. As positive control (C+) staurosporine 1 μM was used. Data represent mean values ± SD from three independent experiments *p < 0.05, **p < 0.01, ***p < 0.001

As shown in Fig. 2a, after 48 h of NPR incubation with the cells, the impact of NPR-PG in terms of ROS production is apparent in half of the cell lines (MEF, MC57G and HeLa cells). However, increasing concentrations of NPR-P induce ROS generation only in MEF cells. The level of ROS generation also depends on the cell type. To be noticed, A549 cells, a highly resistant tumoral cell line widely used for in vitro toxicity studies, show the lowest ROS levels. However they presented a level of NPR internalization similar to the one observed in MEF and HeLa cells, which showed the highest ROS production. The cytotoxic drug staurosporine increases ROS in half of the cell lines although as in the case of NPRs at different levels, indicating that ROS production depends on the cell line. Again, these results provide crucial evidence on the importance of using several cell models to properly interpret the results employing NPs.

### Mitochondrial damage: Loss of mitochondrial membrane potential

ROS generation is mostly a consequence of uncoupled electron mitochondrial transport and usually it is accompanied by a loss of mitochondrial membrane potential. In order to investigate the possible mitochondrial damage caused by NPRs, cells were supplemented with increasing concentrations of NPR-P or NPR-PG. Figure 2b shows the important impact of increasing concentrations of NPRs on the mitochondrial membrane potential (ΔΨ_m_). Although there are differences between the cell lines concerning the loss of ΔΨ_m_, the concentration and functionalization effect is apparent. However, it should be stressed that, when ROS and ΔΨ_m_ data are directly compared for each cell line, there are clear differences between them. For example, loss of ΔΨ_m_ correlates with ROS generation in HeLa, MEF, MC57G and B16 cells; yet the impact of NPR internalization on ROS generation and loss of ΔΨ_m_ is more evident in HeLa and MC57G cells than in B16 cells. In contrast, loss of ΔΨ_m_ does not correlate with ROS generation in A549 cells. Therefore, although the generation of ROS and loss of ΔΨ_m_ correlate with NPR concentration (in particular for glucose-modified NPRs), different cell lines respond differently. TAMRA seemed to have an effect on the loss of ΔΨ_m_ and thus the impact of glucose was difficult to assess in TAMRA functionalized NPR (data not shown). Again staurosporine induced loss of ΔΨ_m_ in all cases except for MC57G cells.

### Apoptosis and NPRs

**Fig. 3:**
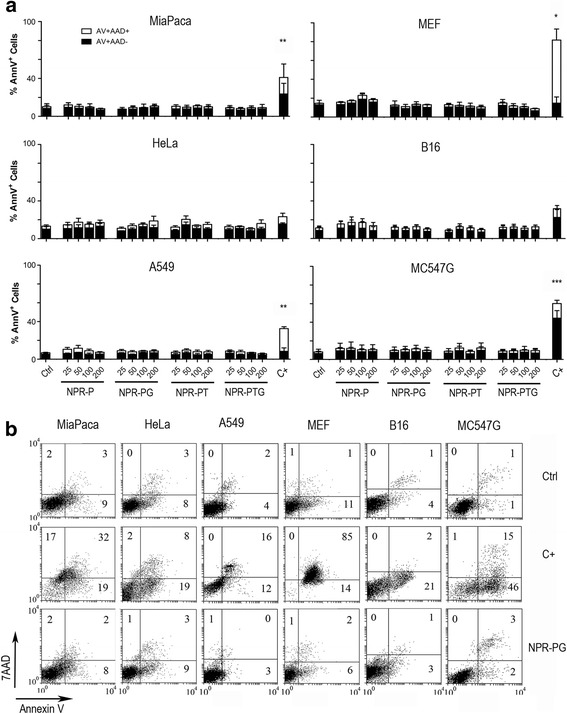
Analysis of phosphatidilserine (PS) translocation and membrane permeabilisation by flow cytometry. The indicated cells were mock treated (ctrl) or incubated with four types of nanoparticles (NPR-P, NPR-PG, NPR-PT, NPR-PTG) at four different concentrations (25, 50, 100 and 200 μg/mL) for 48 h and PS translocation and membrane permeabilisation were monitored using annexin-V and 7AAD respectively as indicated in experimental section. As positive control (C+) staurosporin 1 μM was used. (**a**) Data represent mean values ± SD from three independent experiments. *p < 0.05, **p < 0.01, ***p < 0.001. (**b**). A representative experiment is shown at the concentration of 100 μg/mL. Numbers correspond to the percentage of cells in each quadrant

**Fig. 4:**
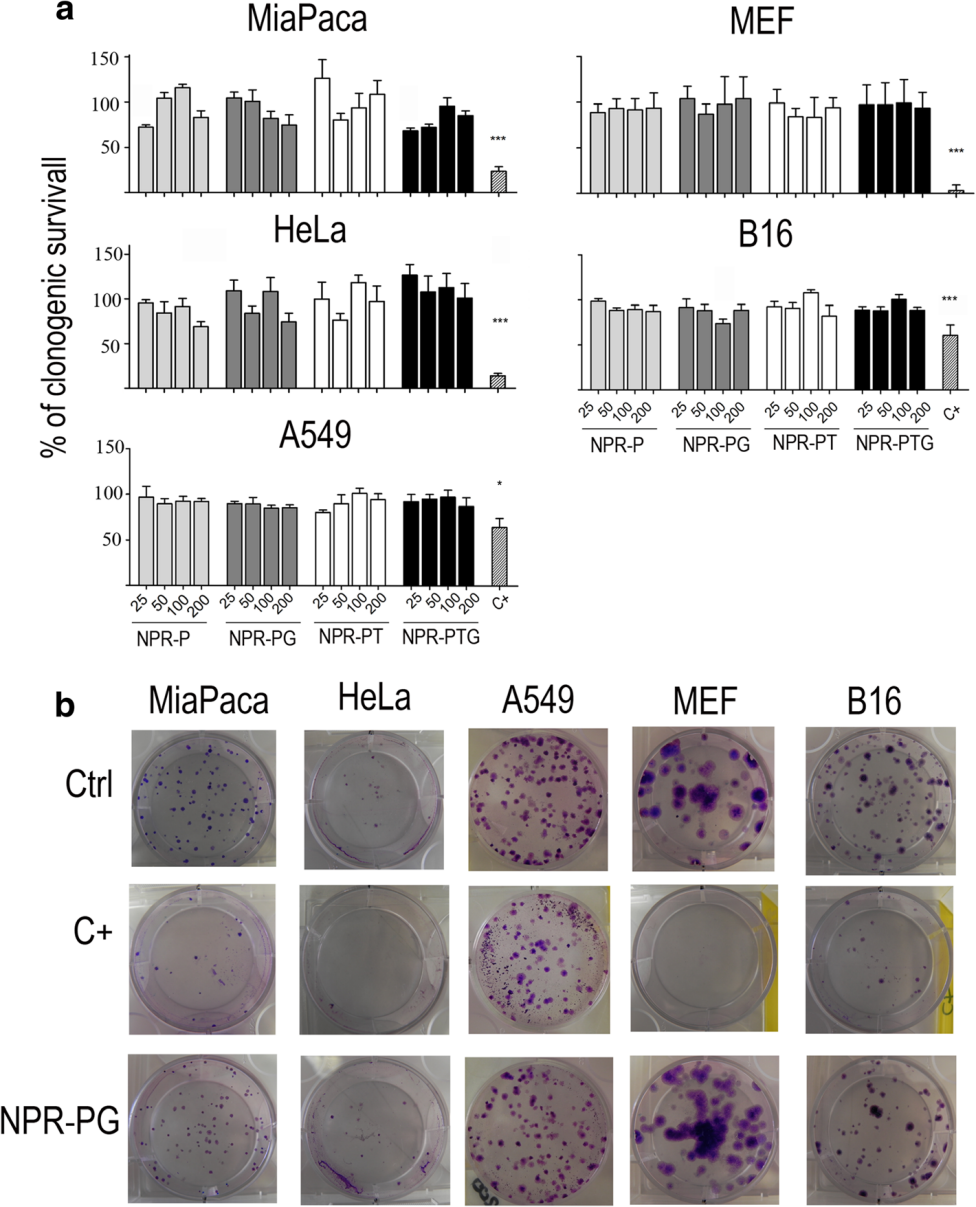
Analysis of the effect of NPRs on cell survival using a clonogenic assay. The indicated cells were mock treated (ctrl) or incubated with four types of nanoparticles (NPR-P, NPR-PG, NPR-PT, NPR-PTG) at four different concentrations (25, 50, 100 and 200 μg/mL) for 48 h. Subsequently, 100 cells were seeded per well by triplicates and incubated in fresh medium for 10 days. After that, cells were stained with Crystal Violet and colonies were counted as described in experimental section. As positive control (C+) staurosporine 1 μM was used. (**a**) Survival was calculated as percentage of colonies growing referred to the number of colonies in the negative control. Data represent mean values ± SD from three independent experiments. *p < 0.05, **p < 0.01, ***p < 0.001. (**b**) Pictures from single wells of a representative experiment (100 μg/mL) are shown

To analyse apoptosis we monitored phosphatydylserine (PS) translocation and membrane damage by flow cytometry using annexin-V and 7-AAD double staining, which allowed us to distinguish between early apoptotic cells (annexinV+/7AAD– cells) and late apoptotic/necrotic cells (annexinV+/7AAD+ cells). As shown in Fig. 3, neither apoptosis nor necrosis was observed in any cell type for any of the NPR derivatives. Staurosporine induced PS translocation in most of the cell lines although at different extent. Although Annexin-V staining did not reach statistically significant difference in HeLa and B16 cells, a clear annexin-V positive cell population could be detected in the dot plots (Fig. 3b) in both cell lines indicating delayed cell death in these cell lines (see Fig. 4).

### Cell proliferation: Clonogenic studies

Finally, to certainly confirm that NPRs did not affect cell viability in vitro, we analysed the effect of NPRs on the ability of the different cell lines to grow and form colonies 10 days after incubation, an assay that is considered as the gold standard to assess cell viability. As shown in Fig. 4, MEF, MiaPaca, HeLa, B16 and A549 cells incubated with the highest concentration of the four NPRs probes form the same number of colonies as the controls. Consequently cell growth and capability of forming colonies (cell viability) are two crucial factors that are not influenced by the incubation of cells with any of the four NPR probes. In contrast, cells incubated with staurosporine showed low proliferation rates in all cell lines, despite the different sensitivity in terms of ROS production and loss of ΔΨ_m_ observed between them. These results prove that staurosporine is able to induce cell death in all cell lines, but with a delayed kinetics in B16 and HeLa cells (compare with Fig. 3a).

This test cannot be applied to all cell lines (MC57G) because of the intrinsic ability of specific cells to form colonies; however, this finding undoubtedly confirms that ROS and mitochondrial changes after NPR exposure are not toxic for the tested cells, including human and mouse cells of tumoral (HeLa, MiaPaca, A549, B16) and non-tumoral (MEF) origin.

### Primary cells: Macrophages and PBMCs

**Fig. 5:**
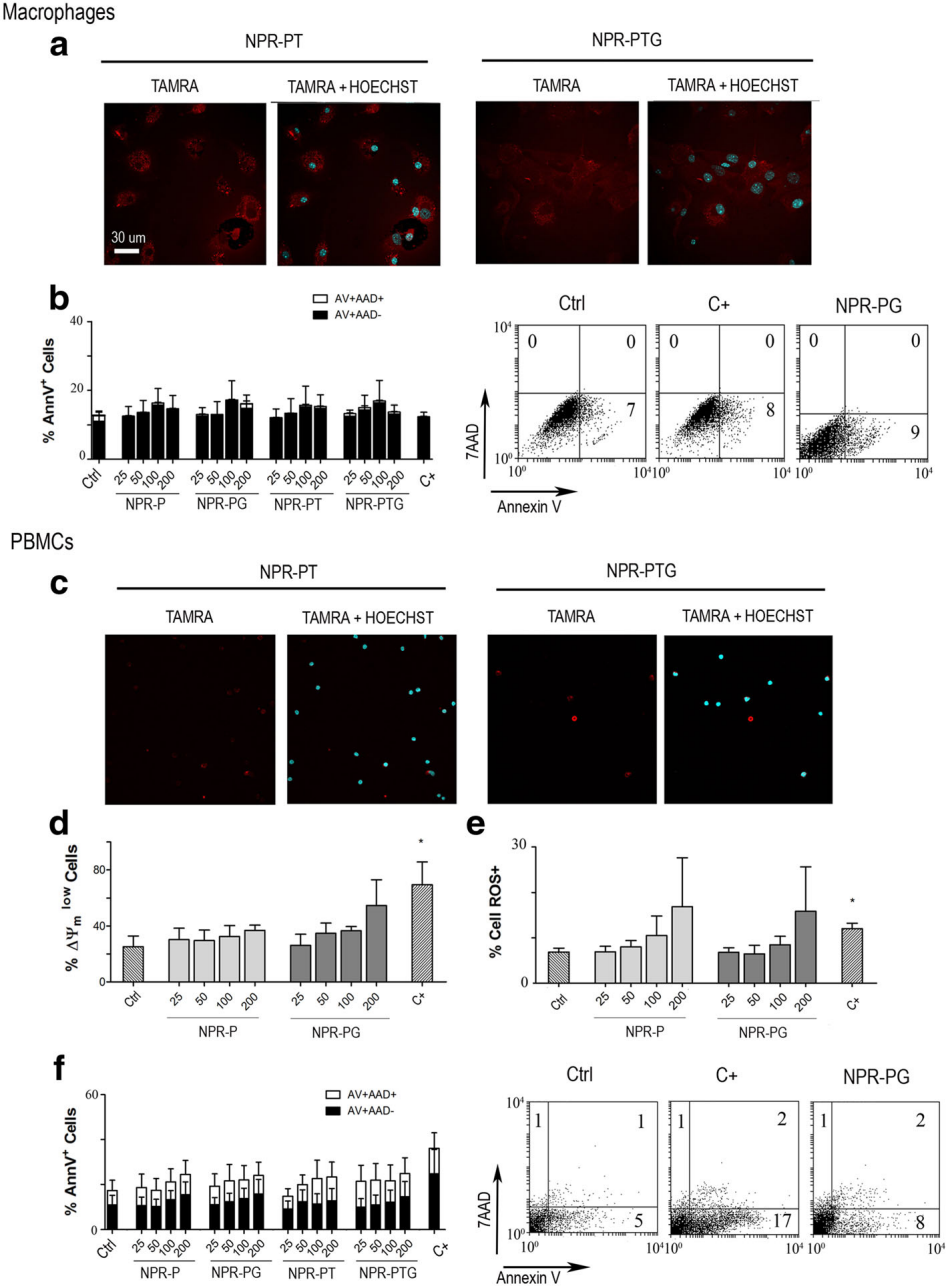
Analysis of the effect of nanoparticles on the viability of mouse primary macrophages and human PBMCs. Mouse bone marrow derived macrophages and human PBMCs were mock treated (ctrl) or incubated with four types of nanoparticles (NPR-P, NPR-PG, NPR-PT, NPR-PTG) at four concentrations (25, 50, 100 and 200 μg/mL) for 24 h as indicated in experimental section. (**a**) Analysis of nanoparticles entry in macrophages using confocal microscopy. A representative experiment 100 μg/mL of NPR-PTG and 200 μg/mL of NPR-PT is shown. (**b**) Detection of phosphatydylserine translocation (AnnexinV) and cell membrane permeabilisation (7AAD) in macrophages by flow citometry. (**c**). Analysis of nanoparticles entry in PBMCs using confocal microscopy. A representative experiment 100 μg/mL of NPR-PTG and 200 μg/mL of NPR-PT is shown. (**d**). Analysis of ΔΨ_m_ loss (DIOC_6_), (**e**) detection of superoxide anion generation and (**f**) detection of phosphatydylserine translocation (AnnexinV) and cell membrane permeabilisation (7AAD) in PBMCs by flow citometry. Data represent mean values ± SD from three independent experiments. *p < 0.05, **p < 0.01, ***p < 0.001. As positive control (C+) staurosporine 1 μM for macrophages and cladribine 5 μM for PBMCs was used. Scale bar: 30 μm

The experiments performed above indicate that gold NPRs are neither cytotoxic nor cytostatic for transformed cells. However this does not necessarily mean that they would not present toxicity in the context of a physiological setting where they encounter with “healthy” cells. To this goal the uptake in primary cells was assayed using the same protocols as in the immortalized cell lines. We chose cells from the immune system (blood lymphocytes and differentiated macrophages) since they are the first ones encountered by NPs when administered in vivo. In the case of bone marrow derived macrophages that presented a inflammatory M1 phenotype (see methods), confocal microscopy confirmed internalization of both NPR-PT and NPR-PTG probes (Fig. 5a)*.* Analysis of ROS generation and loss of ΔΨ_m_ suggested that both processes were induced by all types of NPRs (data not shown). Unfortunately a detailed and reliable quantification of those processes was not possible due to the high level of intrinsic autofluorescence of the macrophages, which is quenched by NPRs. Despite this technical problem, determination of PS translocation (annexin V) and membrane permeabilisation (7AAD) (Fig. 5b) indicated that NPRs are not toxic to the macrophages. Although staurosporine was not able to kill the macrophages as analysed by the annexin V staining, this was not due to an inherent inability to translocate PS since other stimuli like cytotoxic T cells or bacterial infection induced PS translocation in this cell type correlating with loss of cell viability (data not shown and [33]).

In contrast to macrophages and immortalised cell lines, confocal microscopy pictures show that the NPR-PT and NPR-PTG probes displayed a poor internalization by human blood lymphocytes (PBMCs) (Fig. 5c). ΔΨ_m_ and ROS generation were less affected than in cell lines, likely due to the lower internalization rate observed here (Fig. 5d and e) and, again, PS translocation and membrane permeabilisation were absent (Fig. 5f), indicating that NPRs were neither toxic for lymphocytes.

### In vivo toxicity studies

**Fig. 6:**
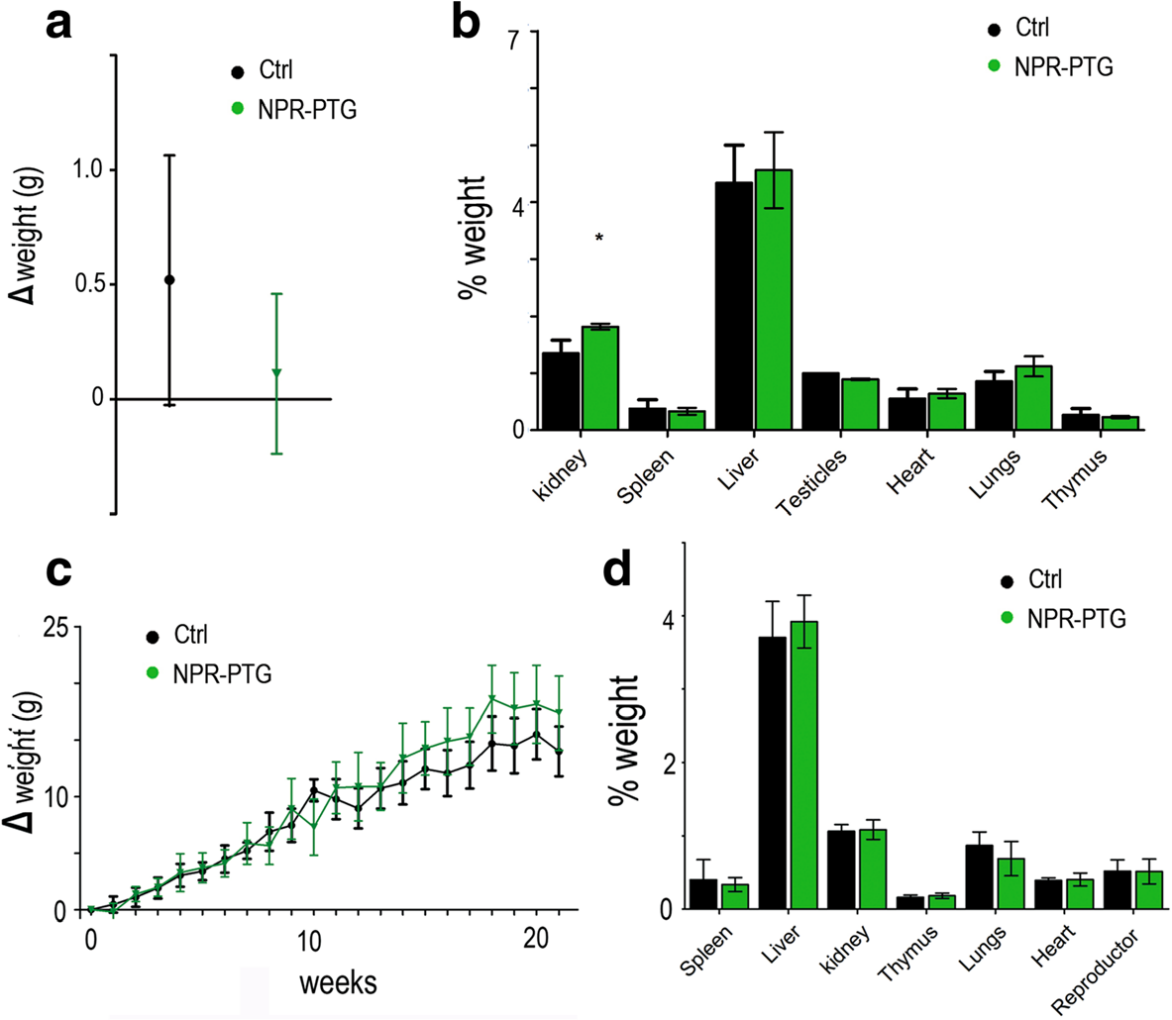
Analysis of (**a** and **c**) the weight gain of mice treated with nanoparticles and (**b** and **d**) the relative weight of the organs after treatment. Data of (**a** and **b**) 3 days and (**c** and **d**) 4 months after treatment. Mice were injected (i.v) with 6 μg/g NPR-PTG (green) or the same volume of PBS in the group control (black). The mice weight was measured (**a**) before and after 3 days of the treatment and (**c**) weekly during the following 4 months. (**b**) and (**d**) the mice were sacrificed and the weight of the different organs were measured after treatment and referred to the mice weight. Data represent mean values ± SD from 5 mice. *p < 0.05

**Fig. 7:**
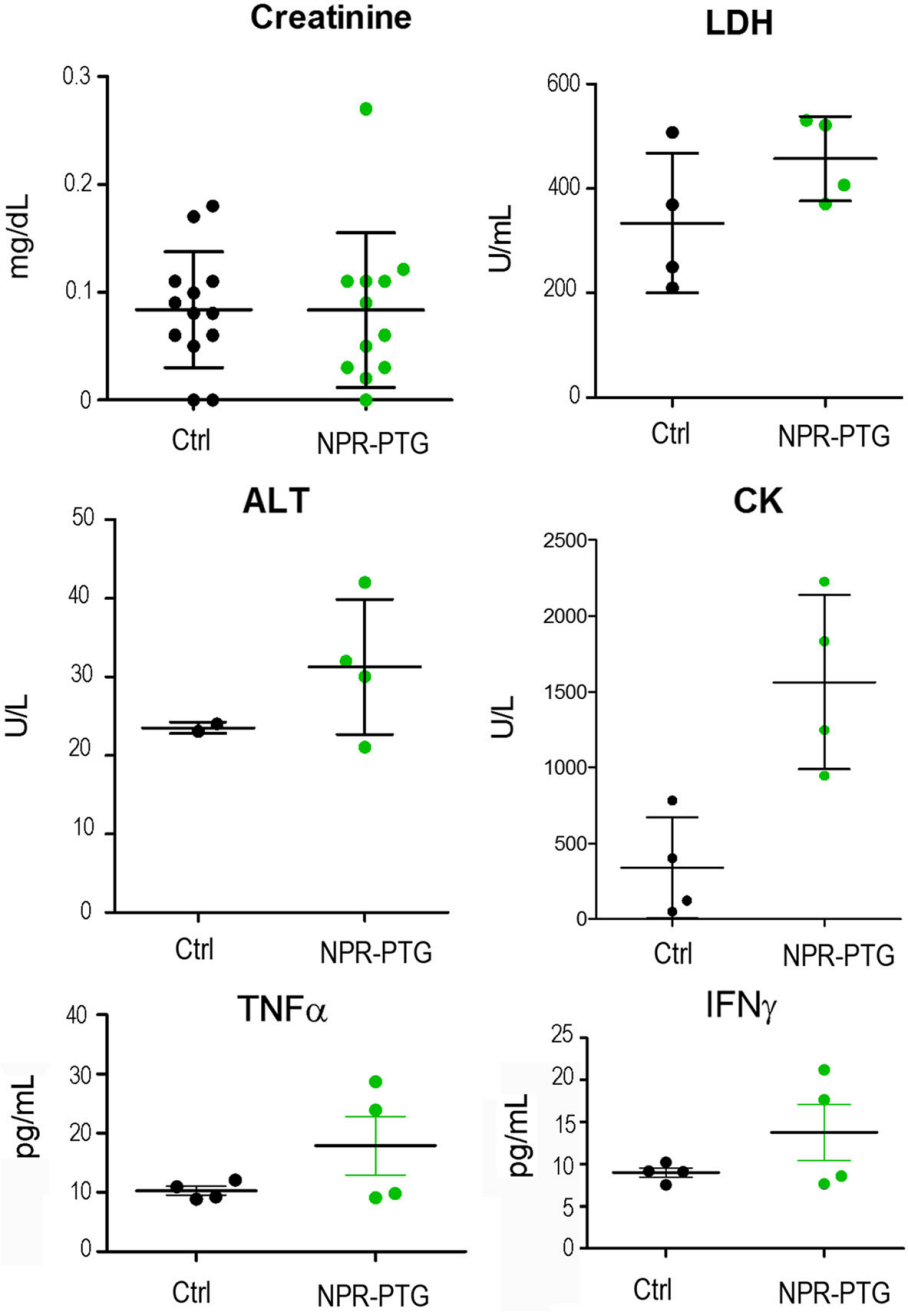
Analysis of four blood biochemical parameters of systemic or specific organ damage (ALT –liver-, CK –muscle-, creatinine –kidney- and LDH –any tissue/cell-) and two main inflammatory cytokines (TNFα and IFNγ). Mice were injected (i.v) with 6 μg/g NPR-PTG (green) or the same volume of PBS (black) in the group control. The mice were sacrificed after four months and the blood was collected by cardiac punction as indicated in experimental section. Dots represent values from each mouse and bars ± SD

Intravenous administration of 6 μg/g NPR-PTG did not affect animal body weight 72 h or 4 months after administration (Fig. 6a and c). Neither other signs of toxicity or animal distress such as disheveled hair, irregular respiration, gastrointestinal symptoms, immobility or death were observed. After extracting the main organs, differences in organ index were not detected, except for kidneys 72 h after the NPs injection, which appeared bigger when compared to those of control mice (Fig. 6b). There were no observable differences in organ indexes four months after injection (Fig. 6d). Among four blood biochemical parameters of systemic or specific organ damage (ALT –liver-, CK –muscle-, creatinine –kidney- and LDH –any tissue/cell-) and two main inflammatory cytokines (IFNγ and TNFα as markers of innate and adaptative inflammatory responses) that were analyzed no significant variations were found, indicating that NPRs did not induce any detectable tissue or cell damage, including inflammatory response (Fig. 7).

**Fig. 8:**
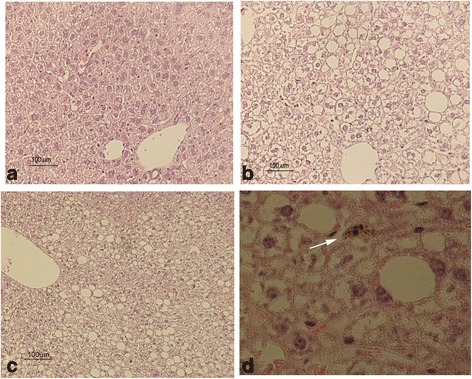
Hematoxylin-Eosin stain of the liver of mice treated with NPRs. Mice were injected (i.v) with (**b**-**d**) 6 μg/g NPR-PTG or the same volume of PBS in the (**a**) group control. The mice were sacrificed after four months and organs were fixed and processed for the H&E stain, as indicated in experimental section. Representative images are shown

**Fig. 9:**
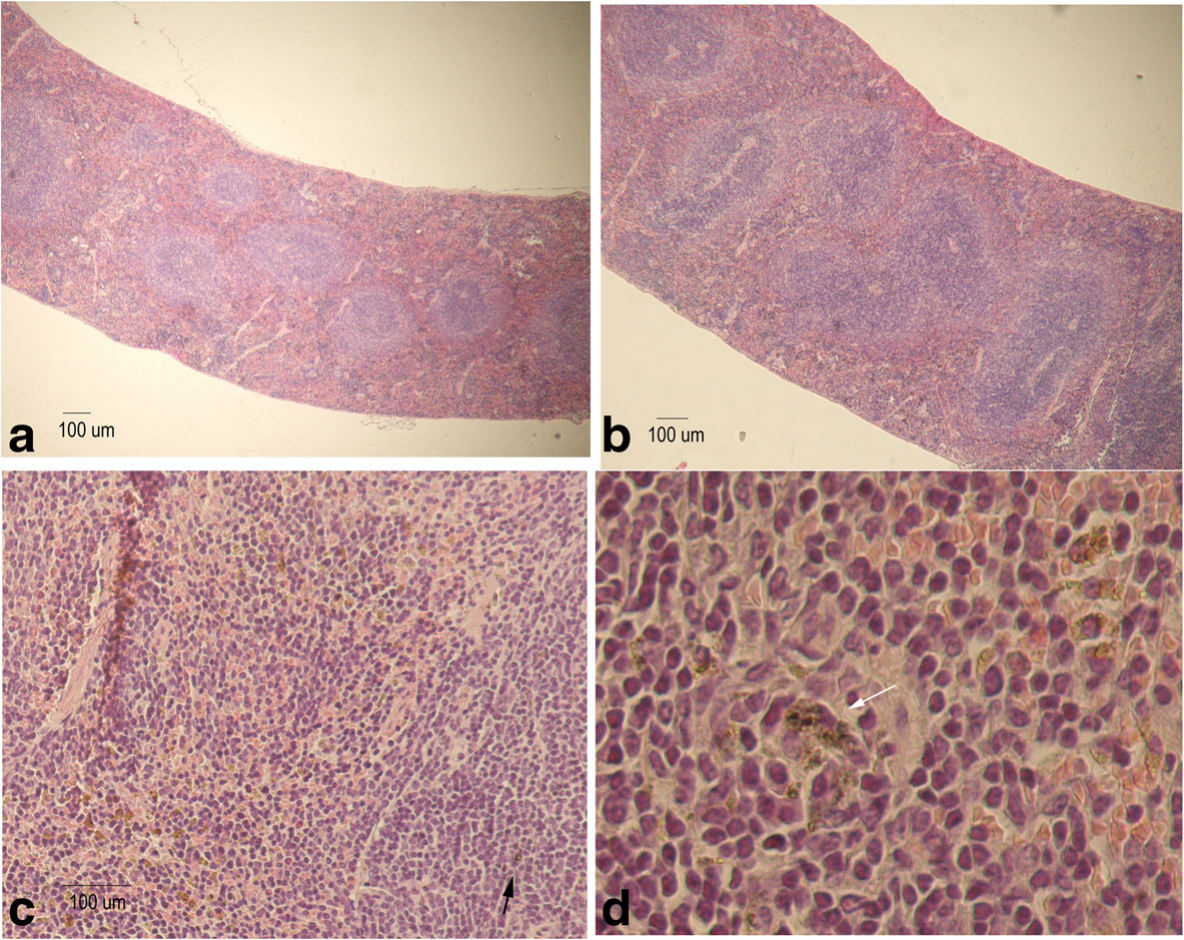
Hematoxylin-Eosin stain of the spleen of mice treated with nanoparticles. Mice were injected (i.v) with (**b**-**d**) 6 μg/g NPR-PG or the same volume of PBS in the (**a**) group control. The mice were sacrificed after 4 months and the organs were fixed and processed for the H&E stain, as indicated in experimental section. Representative images are shown

Next we analysed tissue sections by haematoxylin/eosin (HE) staining to search for structural changes or apparent histopathological abnormalities. After 72 h no significant changes were found, in any of the analyzed tissues including liver, spleen, kidney, heart, lung, thymus and reproductive organs. In turn, four months later, hepatocytes were enlarged and displayed a high number of vacuoles in their cytoplasm (Fig. 8). These changes appeared more frequently in the central lobular section, being compatible with a hydropic change or vacuolar degeneration. The presence of glucogen deposits was discarded as analysed employing a glucogen specific dye (Additional file 1: Figure S6). Also, the presence of Kupffer cells with an enlarged cytoplasm was observed. These cells contained a black pigment that could be compatible with the presence of NPR-PTG (Fig. 8c). Although the white pulp of the spleen was hypertrofic, the architecture of the organ was not altered in a considerable way (Fig. 9b). Macrophages with a black pigment in the cytoplasm (compatible with the NPR-PTG) were identified in the white pulp (Fig. 9c and d).

### Biodistribution of the NPRs in vivo

**Fig. 10:**
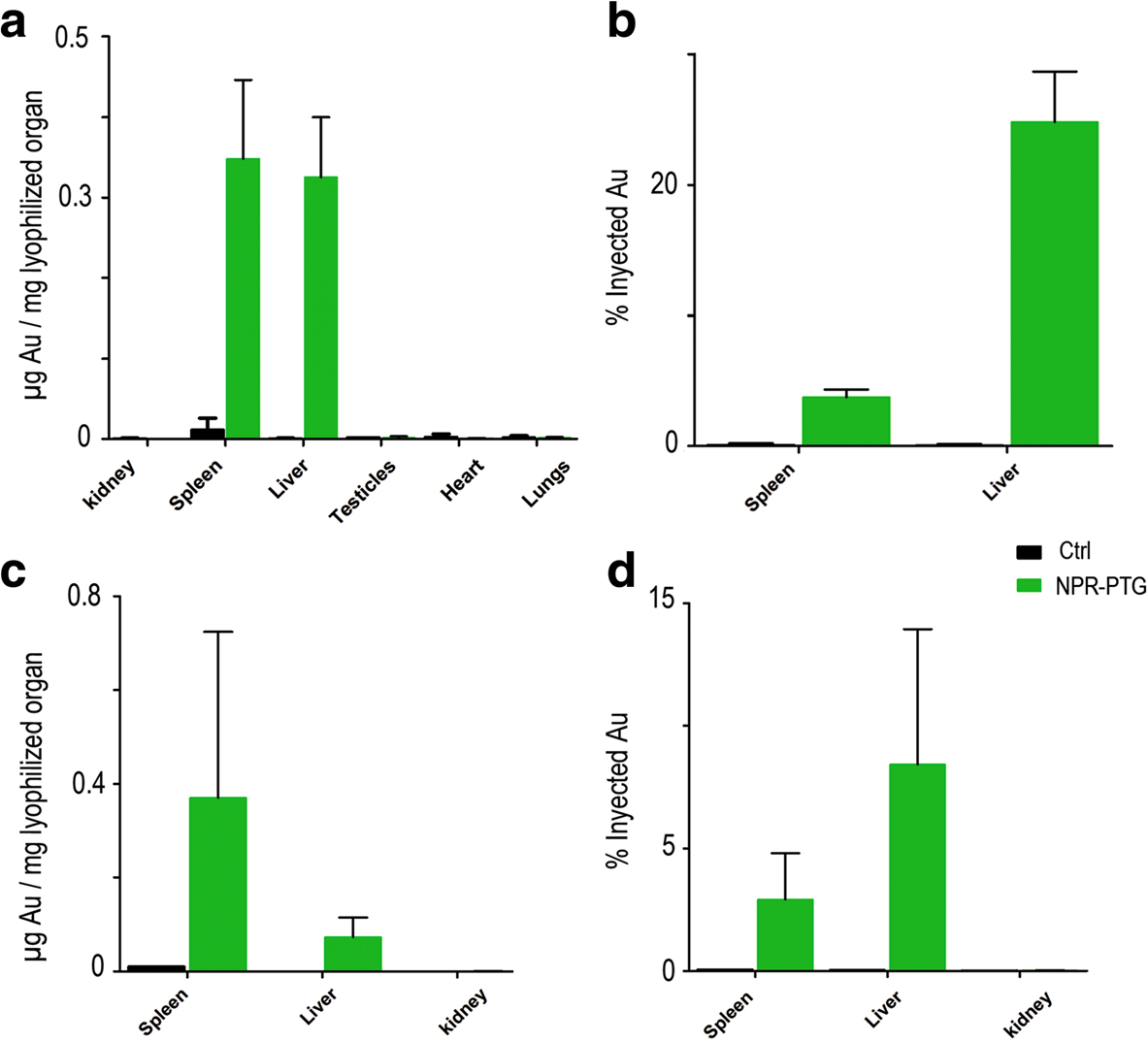
Biodistribution of nanoparticles in vivo. Mice were injected (i.v) with 6 μg/g NPR-PG (green) or the same volume of PBS in the group control (black). The mice were sacrificed after (**a** and **b**) 3 days or (**c** and **d**) four months and the organs were lyophilized and processed as indicated in experimental section, in order to analyse the quantity of gold by ICP-MS. Data represent mean values ± SD from three mice

Fluorescence microscope studies were carried out to determine if the NPRs bearing a fluorophore were present in the tissue sections of the main organs, i.e. liver, spleen, kidneys, heart, lungs, thymus and reproductive organs. 72 h after the intravenous injection it was not possible to find in any trace of a fluorescence signal in these organs; but it was identified in the urine collected 24 h after injection. Importantly however, the ICP-MS analysis did not detect gold in urine (data not shown) thereby suggesting that the fluorophore was detached from NPRs, which is in accordance with previous work [34]. Thus, to corroborate the biodistribution of the NPRs, ICP-MS of the organs was carried out. 72 h after treatment NPRs were found in the spleen and liver, approximately 0.4 μg Au *per* mg of lyophilized organ. The amount of NPRs found in the liver corresponded to 25% of the total amount of NPRs originally injected; whereas the spleen contained just 5%. No NPRs were detected in other organs or in the urine (Fig. 10). Note that the organs that were collected are the ones that more frequently accumulate NPs (spleen, liver, lungs) and other organs essential for other vital functions, such as the reproductive organs and thymus were also collected. The remaining NPRs might therefore be contained in other areas not collected, such as the intestines and canvas or be excreted in the faeces.

Four months after the injection, NPR-PTG were still present in liver and spleen. While in the liver the NPs amount was reduced to 10–15% of the injected dose, in the spleen this amount remained similar to that of 72 h post injection (3–5%) suggesting that the liver was somehow able to degrade at least in part the NPR-PTG.

**Fig. 11:**
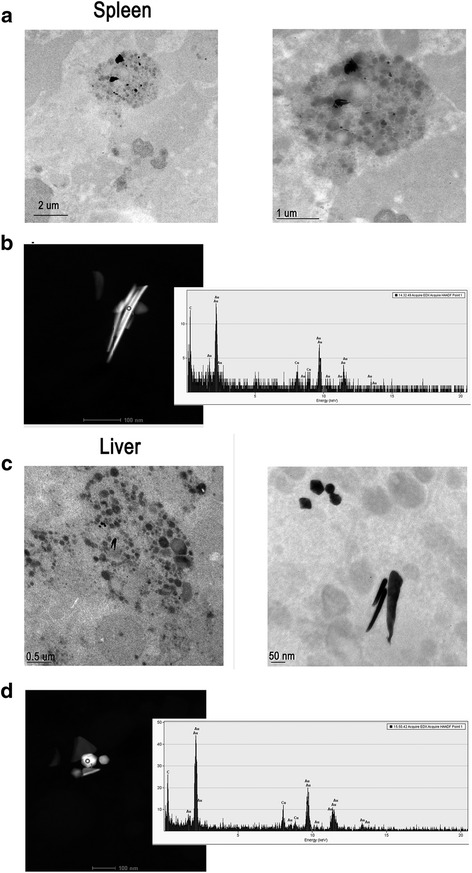
Electronic microscopy analysis of the nanoparticles in the spleen (**a**-**b**) and liver (**c**-**d**) of treated mice. Mice were injected (i.v) with 6 μg/g NPR-PG. Mice were sacrificed after 4 months and the organs were fixed and processed for the TEM (**a** and **c**) and STEM (**b** and **d**) analysis, as indicated in experimental section. Representative images are shown

To confirm the presence of the NPR-PTG in the spleen and liver four months after injection, TEM and STEM along with EDX were performed (Fig. 11). As expected, NPR-PTG were found in macrophages, however, the shape of the NPR-PTG in the liver was different from that of the original NPR, which can be related to a digestion process.

## Discussion

Toxicity is a major concern that needs to be carefully addressed before a new nanomaterial can be considered to be translated into in vivo biomedical clinical application. Moreover, it should be tested at different levels including cell uptake and pharmacokinetics [35], immune/inflammatory responses [36], genotoxic effects or cell death. The overall aim of this work was to investigate the toxicity of anisotropic gold nanoprisms (NPRs) and the influence of the surface coating, focusing on a detailed analyses of its impact on oxidative stress and cell death in vitro as well as toxicity and biodistribution in vivo. To this aim we have employed in vitro cell models and validated the results in vivo in mice. We used sulphate-capped NPRs with ca. 150 nm in length and 10 nm thicknesses (see Additional file 1: Figure S1) modified with PEG. PEG molecules are considered to be among the most efficient antifouling agents used for stabilizing NPs for bioapplications; especially the high molecular weight derivatives such as the 5 kDa chain used in this study [37]. Confirming it, all tests performed on the final modified NPRs proved that the NPRs retain their colloidal stability in the conditions used for the cell studies (see Additional file 1: Figure S4).

We show that cellular internalization of NPRs varies for different cell lines, which may be explained by the various different intrinsic phagocytic properties and/or levels of glucose requirement. Upon confirming the internalization of NPRs by all cell lines, the possible toxic effects induced on those cell types were examined. The pioneering work of Nel and co-workers has established ROS generation as among one of the most common effects caused by internalized NPs [17], although it remains unclear precisely which properties of NPs contribute to the ROS induction. Several studies have focused on surface characteristics, size, shape and other parameters related to increased cytotoxicity by ROS production [20, 38, 39]. A recent study performed on differently functionalized gold and silver NPs highlights also the importance of the components of cell culture medium like serum protein, which upon adsorbtion onto the NPs surface (so-called protein corona) may change the surface charge of NPs and therefore affect their ability to produce ROS [40].

Our results regarding ROS generation indicate that the association of cells with NPRs induced ROS generation in half of the cell lines tested, which was accompanied by loss of ΔΨ_m_ in some cases. Intracellular ROS accumulation can trigger diverse modalities of cell death including apoptosis and necrosis [41]. In line with our findings Soenen et al. found ROS generation and loss of ΔΨ_m_ in a multiparametric study with polymer coated gold NPs of 4 nm in diameter and at concentrations above 100 nM (concentration of gold ca. 40 μg/mL) [22]. However in this case, internalization of those NPs caused DNA damage and cell cytotoxicity. Loss of ΔΨ_m_ and cytotoxicity were prevented by using a ROS scavenger. In contrast to these findings a recent work has highlighted the role of mitochondrial depolarization in tumoral cells, following uptake of positively charged gold NPs as a defense mechanism for counteracting the increased levels of cytosolic Ca^2+^ induced by NP uptake [42]. In line with these findings we show that ROS generation and mitochondrial perturbation are not sufficient to cause apoptosis even in primary cells. Remarkably, NPRs do not induce apoptotic or necrotic features even in cells where the ΔΨ_m_ was down-regulated. In contrast, loss of ΔΨ_m_ and/or ROS generation induced by staurosporine (or other stimuli like cytotoxic T cells or bacterial infection in the case where staurosporine is not effective –data not shown-) were accompanied by induction of phosphatidylserine (PS) translocation and membrane permeabilisation and lower number of colony formation, indicating that lack of pro-apoptotic markers during incubation with NPRs was not due to a general defect in the cells used. Here, it should be noted there are different ways to down-regulate ΔΨ_m_ and not all of them lead to the loss of cell viability. One of them is the formation of a permeability transition pore (PTP) that involves both inner and outer mitochondrial membranes. Its formation is reversible and regulated by intracellular Ca^2+^ and ROS. If cells are able to reduce the levels of ROS and Ca^2+^, the pore is closed and the mitochondrion recovers its function. On the contrary, the permanent opening causes mitochondrial rupture by osmotic shock, triggering cell death [43]. This could help to explain apparent contradictory results observed in different studies. Thus, a reliable measure of toxicity based on ROS production and loss of ΔΨ_m_ will be dependent on the intensity of the stimulus and the ability of the cell to counteract the effect of NPs uptake. Supporting this hypothesis, we have recently shown that the production of high levels of ROS and mitochondrial depolarisation during photothermal therapy using gold NPRs irreversibly lead to cell death [8].

Our data clearly indicate that internalization of NPRs induces signs of cell damage like ROS generation and mitochondrial depolarisation in some cell lines; yet those effects are not sufficient to compromise cell viability as confirmed using clonogeneic assay, the gold standard for analysing cell toxicity. In contrast to the widely used MTT test that only indicates the proliferation status of the cells, a clonogeneic assay tests the ability of single cells to form colonies 10 days after exposure to a specific stimulus [44]. In our case, these assays indicated that NPRs do not affect cell viability at any level including DNA damage or cell cycle arrest. Additionally, this finding indicates that to really assess cellular toxicity of NPs employing in vitro models, several cell types of different origin should be tested using a combination of different assays.

All these results employing transformed cells were confirmed on primary “healthy” cells, such PBMCs and macrophages. NPRs were internalized by macrophages; however, due to the phagocytosis capacities of macrophages no differences were observed in the internalization of different types of NPRs. Mirroring the results obtained with immortalized cell lines, the viability of the macrophages was not compromised. In contrast to macrophages and immortalised cell lines, NPRs were poorly internalized by human PBMCs and thus, ROS generation and ΔΨ_m_ lost were less evident than in the case of the tumour cell lines. Again, NPRs were neither toxic for lymphocytes.

Finally, the absence of toxicity in cell models was confirmed in vivo*,* by treating mice with NPR-PTG and testing the main serum biomarkers of tissue damage and inflammation as well as NP accumulation in organs by anatomy pathology. Although we found that NPRs were accumulated in liver and spleen four months after injection, the animals did not show any sign of mortality or morbidity and all serum and tissue toxicity markers remained unaltered, including those reporting a inflammatory immune response. These results indicate that either in contrast to cell models mitochondrial perturbations are not happening in vivo or if mitochondrial function is also affected in vivo, it is not sufficient to cause acute or chronic toxicity at least during the time frame analysed (four months). Several works have analysed in vivo toxicity of gold nanoparticles with different sizes and forms employing different administration ways [45–48]. A direct comparison with our findings is difficult since administration route, size and morphology (rod, sphere, etc) dramatically change in vivo NP behavior and most of the works have employed gold nanospheres. In these studies it was shown that i.v. administration lead to a liver and spleen accumulation as found here employing NPRs, while oral administration lead to kidney accumulation. Subsequently and in line with our results it was shown that while oral or i.p. administration of gold nanospheres induced toxic effects in mice, i.v. injection did not [47]. In a recent study, 24 h after the injection of 2.7 mg/kg PEGylated NPRs (61.51 ± 2.91 nm) (57 μg per mouse) it was found that the nanoparticles presented the highest level of accumulation in the spleen, whereas the liver and intestine had the next highest concentrations [49]. Moreover, these PEGylated NPRs did not provoke significant biochemical or hematological differences between untreated control mice and nanoparticles-treated mice, which is in accordance to our results.

Gold NPs are traditionally considered inert and thus resistant to degradation in vivo. However, they can be slowly dissolved as cells contain thiols (i.e. glutathione) that can strongly bind to the Au surface, and under certain conditions pull out gold atoms from the NP surface using the surface-bound ligands [50, 51]. Supporting this hypothesis we have observed that although NPR-PTG were not detected in urine by ICP-MS, the fluorophore attached to the surface of the NPR was found just after 24 h. This would mean that the ligand was removed from the NPR once injected and then excreted in the urine. These results are in accordance to those of Kreyling et al. who employing different isotopes to selectively label the core and the shell of AuNPs, found that the Au core was accumulated in the liver, while rest of the coating appeared in urine [34]. The fluorophore excretion could also explain the observed difference in the weight of the kidneys 72 h after injection. Renal cleareance could produce inflammation increasing the relative weight of the kidney [52] which in the present study is a transient effect that could be related with the fluorophore excretion due to the fact that the kidneys recovered their usual weight 4 months after injection.

Together, the quantification of Au by ICP-MS and the results of STEM imaging suggest that liver tissue is able to slowly degrade and eliminate NPRs. In a recent study with iron oxide coated Au nanoparticles it was found that after the dissolution of iron oxide crystals Au particles degraded to form progressively smaller particles that tend to self-assemble together [53]. In a study with 40 nm Au nanoparticles injected i.v. (4.5·10^10^ particles/mouse) it was found that a fraction of gold-loaded cells in the liver gradually decreased to about one fifth after 6 months [54]. Moreover, over time it was found that an increasing part of the total amount of Au nanoparticles in the liver was contained in fewer cells which accumulated in growing clusters. Therefore our observations of small alterations in the volume of the cytoplasm of the hepatocytes and an increase in the number or vacuoles in most of the mice may also suggest ongoing degradation process However, a longer study period (e.g. one year) and more detailed degradation analysis will be required to investigate if NPRs are completely eliminated by liver or in contrast this accumulation results toxic for this organ.

## Conclusions

The physicochemical properties of NPRs highlight them as promising agents for bioapplications including imaging and selective drug delivery. However, the potential secondary effects that could be triggered by NPRs following in vivo administration require special attention. Before performing in vivo toxicity experiments in animal models, the in vitro tests in primary and transformed cells are a must to avoid unnecessary animal usage in case NPRs present high cell toxicity and to design properly the in vivo experiments and the parameters to be tested. Moreover, the effect of NPRs on cellular viability is currently a hotly debated issue that so far has not been clarified. We show here that NPR internalization by a panel of mouse and human transformed and primary cells affect mitochondrial function and ROS generation, yet do not compromise cell viability as shown by clonogenic assays. Thus, our data indicate that assessment of the toxic effects of NPRs in vitro requires of multiparametric analyses of different cell functions before a relevant conclusion could be reached. Other reports have shown that NPR uptake does indeed modify mitochondrial function and generates ROS [22]; however, in some cases these changes compromised cell viability [22, 55, 56] but not in others [24, 42, 57]. In these cases the type of NPRs and cells tested may be the cause of these apparent contradictory findings. Our in vitro results using transformed and primary cells are commensurate with the in vivo findings, which indicate that large (150 nm) gold NPRs are non-toxic when administered intravenously even after four months; although small amounts do accumulate in the liver and spleen and could be associated to small alterations in the liver which nevertheless do not seem to affect the animal health. These are the first analyses of the in vivo toxic effects including inflammatory response of gold NPRs supporting the use of this type of NPs for in vivo biomedical applications. However before gold NPRs could be introduced in clinical settings more detailed studies including other toxicity parameters like a more detailed immunogencity study and genotoxicity and using different coatings, administration routes and prolonged study times will be required.

## Methods

### Synthesis and characterization of the gold NPRs

All chemicals and solvents were of analytical grade purchased from Sigma (Sigma-Aldrich, St. Louis, MO, USA) and used as supplied, without further purification. Pure water was used throughout by passing water through a Milli-Q Academic purification set (Millipore Sigma, Billerica, MA, USA).

To synthesize NPRs, prior to use, all the glass material was washed with *aqua regia* and rinsed thoroughly with MilliQ water. Then, 100 mL of 2 mM (aq.) HAuCl_4_ and 120 mL of freshly prepared 0.5 mM Na_2_S_2_O_3_ were mixed, under mild magnetic stirring conditions for 9 min using c-mag hs stirrer (Ika, Wilmington, NC, USA) (speed 2 of 5 which corresponds to 500 rpm), prior to a second addition of 50 mL of 0.5 mM Na_2_S_2_O_3_. This will produce gold NPRs of ca. 150 nm (146.2 ± 32.4 nm, cf. Fig. S1 in the Additional file 1) of length with possessing a localised surface plasmon resonance (LSPR) band maximum in the LSPR in the range at 1050 nm (NIR range) [7]. These NPRs possess an edge length of ca. 150 nm and height of 10 nm and were characterized by UV-Vis spectroscopy and scanning electron microscopy (Additional file 1: Figure S1) as follows. All samples were measured in aqueous suspension at room temperature using a Varian Cary 50 UV-visible spectrometer (Varian, Corona, CA, USA) over the range of 200–1100 nm in 1 cm quartz cuvettes. Scanning Electron Microscopy (SEM): a single drop of the aqueous solution of the NPRs was placed onto a silicon wafer and SEM analysis were performed using a Inspect F instrument (FEI, Hilsboro, OR, USA) operated at 30 kV. To increase their colloidal stability, NPRs were functionalized with polyethylenglycol (PEG) chain (HS-C_2_H_4_-CONH-PEG-O-C_3_H_6_-COOH) M.W. 5 kDa, (Rapp-Polymer) and resuspended in MilliQ water. In general, 1 mg of thiolated PEG was added to 10 mL of NPRs. Then, the pH of the solution was raised up to 12.0 using 2 M (aq) NaOH. After overnight incubation, NPRs were cleaned of the free PEG by centrifugation (twice for 15 min at 10000 rpm) and the pellet was resuspended in MilliQ water.

NPRs were derivatised with tetramethylrhodamine–5–carboxamide cadaverine (5-TAMRA cadaverine); absorption/emission = 545/576 nm (Anaspec, Fremont, CA, USA), glucose (4-aminophenyl β-D- glucopyranoside) or both ligands. 1 mg NPR were incubated with 1 mg of 1-Ethyl-3-(3-dimethylaminopropyl) carbodiimide hydrochloride (EDC) and 3 mg of N-hydroxysulfosuccinimide (Sulfo-NHS) in 1.5 mL of 50 mM 2-(N-morpholino) ethanesulfonic acid (MES) buffer at pH 6 for 20 min at 37 °C. Activated NPRs were then incubated overnight with 10 μg of TAMRA (NPR-PT); 40 μg of glucose (NPR-PG); or a combination of 10 μg of TAMRA and 40 μg of glucose (NPR-PTG). Functionalized NPRs were then subjected to centrifugal precipitation (twice for 15 min at 10000 rpm) to remove excess reagents and the pellets were resuspended first in MilliQ water.

NPRs were prepared using endotoxin free water and filtered in using 0.22 μm syringe cellulose filters in order to render them sterile. All the experiments were carried out under sterile conditions working with microbiological class II safety cabinets.

NPRs were characterised by UV-Vis and SEM as previous described. Zeta potential measurements were done using a ZetaPALS (Brookhaven, Holtsville, NY, USA). Fluorescence measurements were performed with a LS 55 Fluorescence Spectrometer, 120 V (PerkinElmer, Waltham, MA, USA). Data shown in Additional file 1: Figures. S1-S4.

By using ICP-MS (explained in more detail in animal section) the Au concentration in the stock was quantified. The concentration value was used to determine the extinction coefficient at the maximum of absorbance by plotting the absorbance (at the maximum, LSPR) versus the concentration of different NPs solutions. Therefore, the concentration of the stock solutions could always be estimated by measuring the UV-Vis of the solutions and was always indirectly based in ICP-MS results.

### Cell culture

For cell culture the following reactives were used; DMEM, MEM, RPMI1640, Trypsin-EDTA 0.25% were purchased from PAN biotech (PAN biotech, Aidenbach, Germany). Serum (FBS) (Lonza, Basel, Switzerland). GlutaMAX and pyruvate (Sigma-Aldrich, St. Louis, MO, USA). Penicillin-Streptomycin, non essential aminoacids and sodium bicarbonate (Gibco, Gaithersburg, MD, USA).

SV40 transformed mouse embrionary fibroblasts (MEFs) were kindly provided by Dr. Christoph Borner (Institute of Molecular Medicine and Cell Research, Centre for Biochemistry and Molecular Research, Freiburg) and mouse melanoma (B16) and mouse fibrosarcoma (MC57G) were kindly provided by Markus M Simon (Max-Planck Institute for Immunobiology, Freiburg). Human cervix carcinoma (HeLa), human pancreas carcinoma (MiaPaca) and human lung carcinoma (A549) were acquired from ATCC cultures. MEF, B16, HeLa and MiaPaca cells were cultured in DMEM supplemented with 10% FBS. MC57G cells in MEM supplemented with 10% FBS. A549 cells in DMEM supplemented with 10% FBS, 2.2 g/L NaHCO_3_, 100 mg/L pyruvate and non-essential amino acids. All of them were cultured in an incubator at 37 °C with 5% CO_2_.

Human peripheral-blood mononuclear cells (PBMCs) were obtained from the blood of 3 healthy donors by density gradient centrifugation using Ficoll-Paque (GE-Healthcare, Little Chalfont, UK). Blood samples were provided by blood and tissue bank of Aragón. This study has been approved by the Ethics Committee for Clinical Research of Aragon (CEICA).

Mouse Bone Marrow-derived Macrophages (BMDMs) were differentiated from bone marrow cells as previously described [58]. Briefly, cells were obtained from femurs of 3 C57BL10 mice 6–12 weeks of age by flushing the bone marrow with DMEM and cells were differenciated in BMDM medium (DMEM supplemented with 10% FBS and 5% HS both decomplemented, 2 mM GlutaMAX and 10% of L929 cell culture supernatant as a source of M-CSF) for 6 days. This protocol generates inflammatory CD11b^+^CD11c^−^ macrophages with a M1 phenotype as analysed by its ability to produce IL1β and TNFα in response to LPS and ATP (data not shown).

### Confocal microscopy

2·10^5^ cells were seeded in 24 well plates on 13 mm diameter cover glasses (10^5^ cells/cm^2^) (Electron microscopy science, Hatfield, PA, USA) overnight to allow attachment and treated with 0, 25, 50, 100 and 200 μg/mL NPR-PT and NPR-PTG for 24 h in 1 mL, final volumen. Cells on coverslips were washed with PBS and fixed with 4% paraformaldehyde solution in PBS for 20 min at 4 °C. Fixed cells were mounted on a drop of fluoromount-G (SouthernBiotech, Birmingham, AL, USA) containing 2 μg/mL Hoechst 33,342 (Invitrogen, Carlsbad, USA). Afterward, the cells were analyzed by confocal microscopy (60× objective). Fluorescence images were taken at room temperature on a confocal microscope **(**Olympus FV10-i Oil Type**)** using the same settings for all the cell lines. The software FV10i-SW was used for minor adjustments to background and image overlay.

### Analysis of pro-apoptotic processes by flow cytometry

Cells (2·10^5^ cel/mL) were resuspended in culture medium, seeded at a 10^5^ cells/cm^2^ and treated with the NPRs at different concentrations 0, 25, 50, 100 y 200 μg/mL for 48 h at 37 °C and 5% CO_2_ and the different apoptotic parameters were tested by flow cytometry with a FACS Calibur (BD, Franklin Lakes, NJ, United States) and CellQuests software. ROS generation was analysed using dihydroethidine (2HE, 2 μM) (Invitrogen, Carlsbad, USA), Δψ_m_ was measured with the fluorescent probe 3,3-dihexyloxacarbocyanine iodide (DiOC_6_) 10 nM (Invitrogen, Carlsbad, USA) and PS exposure and 7AAD uptake was analyzed using annexinV–DY634 and 7AAD at 1/100 dilution (Inmunostep, Salamanca, Spain), as follows. Briefly, cells were tripsinyzed, centrifugated 300 xg for 5 min and incubated with the probe for 20 min at room temperature, in cell culture medium for 2HE and DiOC_6_(3) or in annexin binding buffer (Inmunostep, Salamanca, Spain) for annexin V. [58].

### Clonogenic survival assay

Clonogenic assay or colony formation assay is an in vitro cell survival assay based on the ability of a single cell to grow and generate a colony. 48 h after treatment, cells were trypsinized and 100 cells were seeded per well in a final volume of 3 mL in a six-well plate (10 cells/cm^2^). Cells were allowed to grow during 10 days and after that time, medium was removed and cell colonies were counted after fixing and colouring them for 20 min with a mixture of glutaraldehyde (6.0% *v*/v) and crystal violet (0.5% *w*/*v*) at room temperature.

### Animal experimentation

All the experiments involving animals were made according to the law RD53/2013 and approved by the Ethics Committee for Animal Research from University of Zaragoza that is an accreditated animal-welfare body. All the experiments were performed according to the institutional animal use and care regulation of the Centro de Investigación Biomédica de Aragón (CIBA; Zaragoza, Spain). The animals were purchased from the CIBA centre and fed a standard diet ad libitum throughout the experiments. Pathogen-free 6 week old Swiss mice were randomly divided into 2 different groups: mice injected with NPR-PTG and control group where PBS was injected. To reach statistically significant results, 2 independent experiments were performed. Each group (control and treated) consisted of 10 animals, 5 male and 5 female, in order to discard sexual-biased results. NPR-PTG were suspended in 100 μL of PBS and injected intravenously in the tail vein at dose of 6 μg NPR-PTG per g. This dose corresponds to approximately 75–100 μg NPR-PTG/mL blood in the moment of administration, which is in accordance with the concentrations analysed in the experiments in vitro. Mice were sacrificed 72 h and 4 months after the injection in CO_2_ gas chamber. Subsequently the blood has been collected by cardiac puncture using 10% of sodium citrate as anticoagulant for plasma separation via centrifugation (15 min, 2000 x g). ALT (alanine aminotransferase), and CK (creatine kinase), activities was measured according to the IFCC recommendations, by means of the decrease (ALT) or increase (CK) of absorbance at 340 nm. Determination of LDH (lactate dehydrogenase) activity was measured by the transformation of lactate to piruvate, measuring the increase of absorbance at 340 nm. Creatinine concentrations were determined by the Jaffé method. All measurements were carried out using an automated analyzer Cobas c501 (Roche Diagnostics, Indianapolis, IN, USA).

Levels of IFNγ and TNFα were quantified with their respective “Ready-Set-Go” ELISA kit (eBioscience, San Diego, CA USA) following manufacturer’s instructions for which 100 μL serum were needed per each cytokine analyzed in a 96 well plate format. The detection method is based on the enzyme Pre-titrated Avidin HRP (horseradish peroxidase) measuring absorbance at 450 nm and 570 nm in a plate reader Synergy ™ HT (BioTek, Winooski, VT, USA). The results were obtained substrating the values of 570 from those of 450 (Abs _450_-Abs _570_).

Finally liver, spleen, kidneys, heart, lungs, thymus and reproductive organs were excised and prepared for, histopathological tissue examination, TEM, STEM and ICP-MS measurements. In the case of short term study (72 h) three mice from NPR-PTG group and one from control were placed in a metabolic cage enabling urine samples collection every 24 h.

#### Histology examination of haematoxylin and eosin stained tissue sections

For pathological examination of the organs, pieces of the excised organs were fixed with pH 7.4 phosphate-buffered 4% paraformaldehyde during 24 h at 4 °C and processed in mesh cassettes (Mesh Biopsy Cassette) using a standard protocol in an automatic tissue processor; Tissue-Tek Xpress ×50 (Sakura Fineteck, Torrance, CA, USA). The preparation of the blocks was carried out with the block making unit Leica EG1150 (Leica Bioscience, Wetzlar, Germany) after which the blocks were solidified on a cold plate. Samples were embedded in paraffin, sectioned at 3 μm thick (Rotary microtome Leica RM2255) and placed in a tempered bath where they were collected with Superfrost slides (Dako Flex IHC Microscope Slides; ref.: K8020) (Angilent technologies, Santa Clara, CA, USA). The slides were placed in vertical racks and allowed to dry in an oven. After dewaxing and hydration process, the samples were stained with hematoxylin and eosin ready-to-use Dako (Agilent, Santa Clara, CA, USA) and mounted with permanent mounting medium DPX.

#### TEM and STEM characterization of organs

For electron microscopic analysis, the excised organs were cut into 1 mm^3^ pieces and fixed with 2.5% glutaraldehyde/2% paraformaldehyde in 0.1 M sodium phosphate buffer at pH 7.4 for 24 h at room temperature and subsequently stored for 5 days at 4 °C. Thereafter the samples were washed for 30 min four times with sodium phosphate buffer, post-fixed with 2% osmium, rinsed, dehydrated and embedded in Durcupan resin Fluka (Sigma-Aldrich, St. Louis, MO, USA). Semithin sections (1.5 μm) were cut with an Ultracut UC-6 (Leica, Wetzlar, Germany) and stained lightly with 1% toluidine blue. Finally, ultra-thin sections (0.08 μm) were cut with a diamond knife, stained with lead citrate (Reynolds solution) and examined under a 300 kV transmission electron microscope FEI Tecnai F30 enabling working in STEM mode and performing X-Ray Microanalysis (EDS) (FEI Europe, Eindhoven, The Netherlands).

#### ICP-MS

Organs were washed in PBS buffer and frozen in liquid nitrogen upon collection. Following, they were thawed and washed trice in distillate water. All samples were lyophilized using Laboratory Freeze Dryer CRYODOS (Telstar Industrial, S.L., Terrassa, Spain), weighted, placed in porcelain crucibles and burned in a Mini Muffle Furnace (Nabertherm GmbH, Lilienthal, Germany) at 600 °C during 45 min. To the cooled ashes 200 μL of piranha solution (>85% sulphuric acid and > 50% hydrogen peroxide) were added and the solution was left for 10 min. Following, 200 μL of *aqua regia* was added, the solution was transferred to a 15 mL centrifuge tube (TPP Techno Plastic Products AG, Trasadingen, Switzerland) and heated at 70 °C for one hour using a thermoblock (Thermo Fisher, Massachusetts, USA). For ICP analysis, the content of acids has to be between 1 and 5%. Therefore, the samples were centrifuged for 5 min to eliminate the ashes, trasfered to a 50 mL centrifuge tube (TPP Techno Plastic Products AG, Trasadingen, Switzerland) and diluted to the final volume of 20 mL (2% of acids) using graduated cylinder.

The quantitative Au analysis was performed by using ICP-MS 7700× (Agilent Technologies, Sta Clara, CA, USA). ICP-MS was used for Au determination using a MicroMist micro-uptake glass concentric nebulizer (Glass Expansion, West Melbourne, Victoria, Australia). In order to reduce MO^+^ formation in the plasma, the spray chamber was peltier cooled at 2 °C. A standard quartz torch with 2.5 mm internal diameter injector was used. Finally, standard nickel cones (sample and skimmer) were used. The optimization of the ICP-MS conditions was achieved by adjusting the torch position and tuning for reducing oxide and doubly charged ion formation with a standard tuning solution containing 1.0 μg L^−1^ of ^7^Li, ^24^Mg, ^59^Co, ^89^Y, ^140^Ce and ^205^Tl in 1.0% HNO_3_. This equipment includes a collision cell (He gas, ORS3 system, Agilent Technologies ©) for discriminate spectral interferences with high performance for all the trace metals considered in here. The calibration curve comprises a linear range from 0.01 to 20 ppb.

The precision of the analysis is < 10%. Other parameters such as accuracy and recovery could not be determined without knowing the exact Au content in the sample before measuring it.

All the analysis were performed in the Servicio Central Analisis de Bizkaia (Universidad del Pais Vasco).

### Statistical analysis

All data are presented as the mean ± standard deviation (SD). Significant differences between groups were evaluated by two-way ANOVA with Bonferroni post-test using GraphPad Prism 5.0 (GraphPad 339 Software, CA, USA). * *p* < 0.05, **p < 0.01, ***p < 0.001.

## Additional file

Additional file 1:
**Figure S1.** a) SEM micrograph of the initial NPR-P. b) Length (L) distribution of the NPRs, average length is 146.2 ± 32.4 nm. c) UV/Vis spectra of a NPR-P solution. **Figure S2.** a) Agarose gel (0.7%, 100 V, 1 h) of the pegylated NPRs (NPR-P) and the modified ones (NPR-PT, NPR-PTG and NPR-PG). b) ζ-potential measurements of the final NPRs measured in water and using a concentration of 0.04 mg/mL. **Figure S3.** Fluorescence spectra of NPRs solutions labeled with TAMRA. In black, there is the normalized spectrum for NPR-PT, and in red the normalized spectra for NPR-PTG. **Figure S4.** UV/Vis spectra of NPRs before (red) and after (black) their incubation in complete cell media during 24 h. **Figure S5.** (A) Analysis of cell morphology changes by flow cytometry. MiaPaca, HeLa, A549, MEF, B16, MC57G line cells were incubated with four types of nanoparticles (NPR-P, NPR-PG, NPR-PT, NPR-PTG) at four concentrations (25, 50, 100 and 200 μg/mL) for 48 h as indicated in experimental section. After incubation time, cells were analyzed by flow cytometry. A representative experiment is shown at the concentration of 200 μg/mL *Note:* Ctrl = negative control. **Figure S6.** Periodic acid-Schiff (PAS) and periodic acid-Schiff diastasa (PAS/D) stain of the liver of mice treated with NPRs. Mice were injected (i.v) with 6 μg/g NPR-PTG and sacrificed after 4 months, the organs were fixed and processed for PAS staining, as indicated in experimental section. Representative images are shown. (DOCX 45107 kb)

## Abbreviations

2HE: dihydroethidine
AnnV: Annexin-V
NPR: Nanoprisms
NPR-P: PEGylated nanoprisms
NPR-PG: PEGGlylated nanoprisms modified with glucose
NPR-PT: PEGylated nanoprisms modified with rhodamine
NPR-PTG: PEGGlylated nanoprisms modified with glucose and rhodamine
NPs: nanoparticles
PBMCs: Peripheral blood mononuclear cells
PEG: Polyethylene glycol
PS: Phosphatydylserine
PTP: Permeability transition pore
ROS: Reactive oxygen species
SEM: Scanning electron microscopy
SSC: Side-scattered light
ΔΨ_m_: mitochondrial membrane potential
