# Supplementary material for: Multiparametric analysis of anti-proliferative and apoptotic effects of gold nanoprisms on mouse and human primary and transformed cells, biodistribution and toxicity in vivo
Source: Part Fibre Toxicol. 2017 Oct 26;14:41. doi: 10.1186/s12989-017-0222-4 (PMC5658988; doi:10.1186/s12989-017-0222-4)
Supplement: Additional file 1: — Figure S1. a) SEM micrograph of the initial NPR-P. b) Length (L) distribution of the NPRs, average length is 146.2 ± 32.4 nm. c) UV/Vis spectra of a NPR-P solution. Figure S2. a) Agarose gel (0.7%, 100 V, 1 h) of the pegylated NPRs (NPR-P) and the modified ones (NPR-PT, NPR-PTG and NPR-PG). b) ζ-potential measurements of the final NPRs measured in water and using a concentration of 0.04 mg/mL. Figure S3. Fluorescence spectra of NPRs solutions labeled with TAMRA. In black, there is the normalized spectrum for NPR-PT, and in red the normalized spectra for NPR-PTG. Figure S4. UV/Vis spectra of NPRs before (red) and after (black) their incubation in complete cell media during 24 h. Figure S5. (A) Analysis of cell morphology changes by flow cytometry. MiaPaca, HeLa, A549, MEF, B16, MC57G line cells were incubated with four types of nanoparticles (NPR-P, NPR-PG, NPR-PT, NPR-PTG) at four concentrations (25, 50, 100 and 200 μg/mL) for 48 h as indicated in experimental section. After incubation time, cells were analyzed by flow cytometry. A representative experiment is shown at the concentration of 200 μg/mL Note: Ctrl = negative control. Figure S6. Periodic acid-Schiff (PAS) and periodic acid-Schiff diastasa (PAS/D) stain of the liver of mice treated with NPRs. Mice were injected (i.v) with 6 μg/g NPR-PTG and sacrificed after 4 months, the organs were fixed and processed for PAS staining, as indicated in experimental section. Representative images are shown. (DOCX 45107 kb) [file 12989_2017_222_MOESM1_ESM.docx]

**Additional file 1**

**Multiparametric analysis of anti-proliferative and apoptotic effects of gold nanoprisms on mice and human primary and transformed cells**

*Marta Pérez-Hernández*

Department of Biochemistry and Molecular and Cell Biology, Fac. Ciencias, Universidad de Zaragoza, 50009 Zaragoza, Spain and Immune Effector Cells Group, Aragón Health Research Institute (IIS Aragón), Biomedical Research Centre of Aragón (CIBA), Universidad de Zaragoza, 50009 Zaragoza, Spain

martaperezh@gmail.com

*María Moros*

Instituto Universitario de Nanociencia de Aragón (INA), Universidad de Zaragoza, 50018 Zaragoza, Spain and Institute of Applied Sciences and Intelligent Systems-CNR, Via Campi Flegrei, 34, 80078, Pozzuoli, Italy

m.moros@isasi.cnr.it

*Grazyna Stepien*

Fundación Instituto Universitario de Nanociencia de Aragón (FINA), Universidad de Zaragoza, 50018 Zaragoza, Spain and CIBER in Bioengineering,
Biomaterials and Nanomedicine (CIBER-BBN)

g.el.stepien@gmail.com

*Pablo del Pino*

Instituto Universitario de Nanociencia de Aragón (INA), Universidad de Zaragoza, 50018 Zaragoza, Spain, and Centro Singular de Investigación en Química Biológica y Materiales Moleculares (CiQUS) y Departamento de Física de Partículas, Universidade de Santiago de Compostela, 15782 Santiago de Compostela, Spain

pablo.delpino@usc.es

*Scott G. Mitchell*

Instituto de Ciencia de Materiales de Aragón, CSIC-Universidad de Zaragoza and CIBER in Bioengineering, Biomaterials and Nanomedicine (CIBER-BBN)

scott@unizar.es

*Sebastián Menao*

Department of Clinical Biochemistry. H.C.U. Lozano Blesa, Zaragoza 50009, Spain.

smenao@unizar.es

*Marcelo de las Heras*

Department of Animal Pathology, Veterinary Faculty, University of Zaragoza, 50009 Zaragoza, Spain

lasheras@unizar.es

*Beatriz Pelaz*

Instituto Universitario de Nanociencia de Aragón (INA), Universidad de Zaragoza, 50018 Zaragoza, Spain, and Centro Singular de Investigación en Química Biológica y Materiales Moleculares (CiQUS) y Departamento de Física de Partículas, Universidade de Santiago de Compostela, 15782 Santiago de Compostela, Spain

beatriz.pelaz@usc.es

*Maykel A. Arias*

Immune Effector Cells Group, Aragón Health Research Institute (IIS Aragón), Biomedical Research Centre of Aragón (CIBA), Universidad de Zaragoza, 50009 Zaragoza, Spain

maykelariascabrero@gmail.com

*Eva M Gálvez*

Immune Effector Cells Group, Aragón Health Research Institute (IIS Aragón), Biomedical Research Centre of Aragón (CIBA), Universidad de Zaragoza, 50009 Zaragoza, Spain and Instituto de Carboquímica ICB-CSIC, 50018 Zaragoza, Spain

eva@icb.csic.es

*Jesús M. de la Fuente**

Instituto de Ciencia de Materiales de Aragón, CSIC-Universidad de Zaragoza and CIBER in Bioengineering, Biomaterials and Nanomedicine (CIBER-BBN)

jmfuente@unizar.es

*Julián Pardo **

Instituto Universitario de Nanociencia de Aragón (INA), Universidad de Zaragoza, 50018 Zaragoza, Spain, Biomedical Research Center of Aragón (CIBA), Aragón Health Research Institute (IIS Aragón), University of Zaragoza, 50009 Zaragoza, Spain and Department of Microbiology, Preventive Medicine and Public Health, Medicine School, University of Zaragoza, 50009 Zaragoza, Spain

Aragón I+D Foundation (ARAID), Government of Aragon, Zaragoza, Spain

pardojim@unizar.es

* Corresponding author

**1: Characterization of NPRs**

UV/visible absorption measurements were carried out with a Varian Cary 5 UV/vis/NIR spectrophotometer at room temperature. The sample grids for SEM measurements were prepared by dropping an aqueous suspension of NPRs onto a silicon wafer. Grids were imaged using a field emission FEI Inspect F instrument operated at 30 kV scanning electron microscope (SEM). ζ-potential measurements were performed using a nanosizer (Malvern). Fluorescence measurements were performed with a LS 55 Fluorescence Spectrometer, 120V.

NPRs were synthesized and stabilized with PEG (NPR-P) then fully characterized, as previously described [1]. For the interested reader about intracellular location of NPRs in cell cultures, we refer to recent work, [2] which showed that NPRs are stored in endocytic vesicles (endosomes/lysosomes).

Figure S1 shows in panel a) a scanning electron micrograph of the final NPRs and the corresponding histogram (b). Since many of them are laying on their edges it is possible to measure the length (L) and the width of the NPRs. In the UV/Vis spectrum c) the maximum at *ca.* 1050 nm corresponds to the localized surface plasmon resonance absorbance of the NPRs in the NIR region, located in the biological window. The spectrum also indicates that the sample composition was mostly triangular nanoprisms, which is in agreement with the image obtained by SEM.

Figure S1. a) SEM micrograph of the initial NPR-P. b) Length (L) distribution of the NPRs, average length is 146.2 ± 32.4 nm. c) UV/Vis spectra of a NPR-P solution.

UV/Vis spectra showed no significant change compared with the initial ones (data not shown).^1^ Modified samples were run into an agarose gel 0.7 % using 100 V for 1 h (Figure S2 a). The increased stability that the PEG provide them allow to the NPRs to run into the gel matrix. After their surface modification final NPRs showed a different electrophoretical mobility. This difference is due the change of their surface charge which was corroborated by ζ-potential measurements in water finding less negative values for the modified NPRs as expected (Figure S2 b).

Figure S2. a) Agarose gel (0.7 %, 100 V, 1 h) of the pegylated NPRs (NPR-P) and the modified ones (NPR-PT, NPR-PTG and NPR-PG). b) ζ-potential measurements of the final NPRs measured in water and using a concentration of 0.04 mg/mL.

The fluorescence spectrum of the NPRs was determined using as excitation wavelength 545 nm. Maximum in both cases (Figure S3, NPR-PT and NPR-PTG) was found at 576 nm, which is in agreement with the wavelengths provided for the 5-tamra-cadaverine dye.


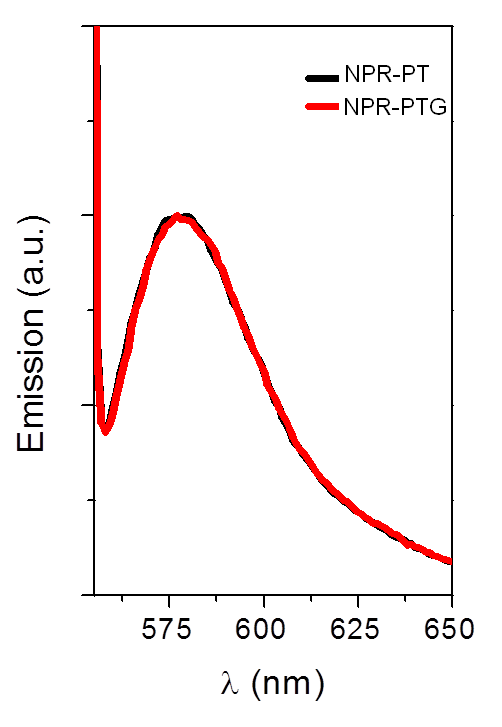


Figure S3. Fluorescence spectra of NPRs solutions labeled with TAMRA. In black, there is the normalized spectrum for NPR-PT, and in red the normalized spectra for NPR-PTG.

The stability of the NPRs in cell media was evaluated using UV/Vis spectroscopy. In Figure S4, the UV-vis spectra of NPRs before (red) and after (black) their incubation in complete cell media (Dulbecco’s modified Eagle’s medium (DMEM) supplemented with 10% foetal bovine serum (FBS), 1% L-Glutamine, and 1% penicillin/streptomycin) during 24 h are shown. Samples were washed three times with water by centrifugation (20 min, 8000 rpm). No significant differences were found between spectra, which demonstrate that NPRs are stable in the medium (at least for 24 h), *i.e.,* they remain colloidally stable with no sign of aggregation.

Figure S4. UV/Vis spectra of NPRs before (red) and after (black) their incubation in complete cell media during 24h.

**2: Morphology assessment.**

The uptake of NPRs by different cell lines (as well as their relative NPR’s uptake) was confirmed by a concentration-dependent increase in the mean intensity of the side scatter channel (SSC) in FACS analysis, *cf.* Figure S5.


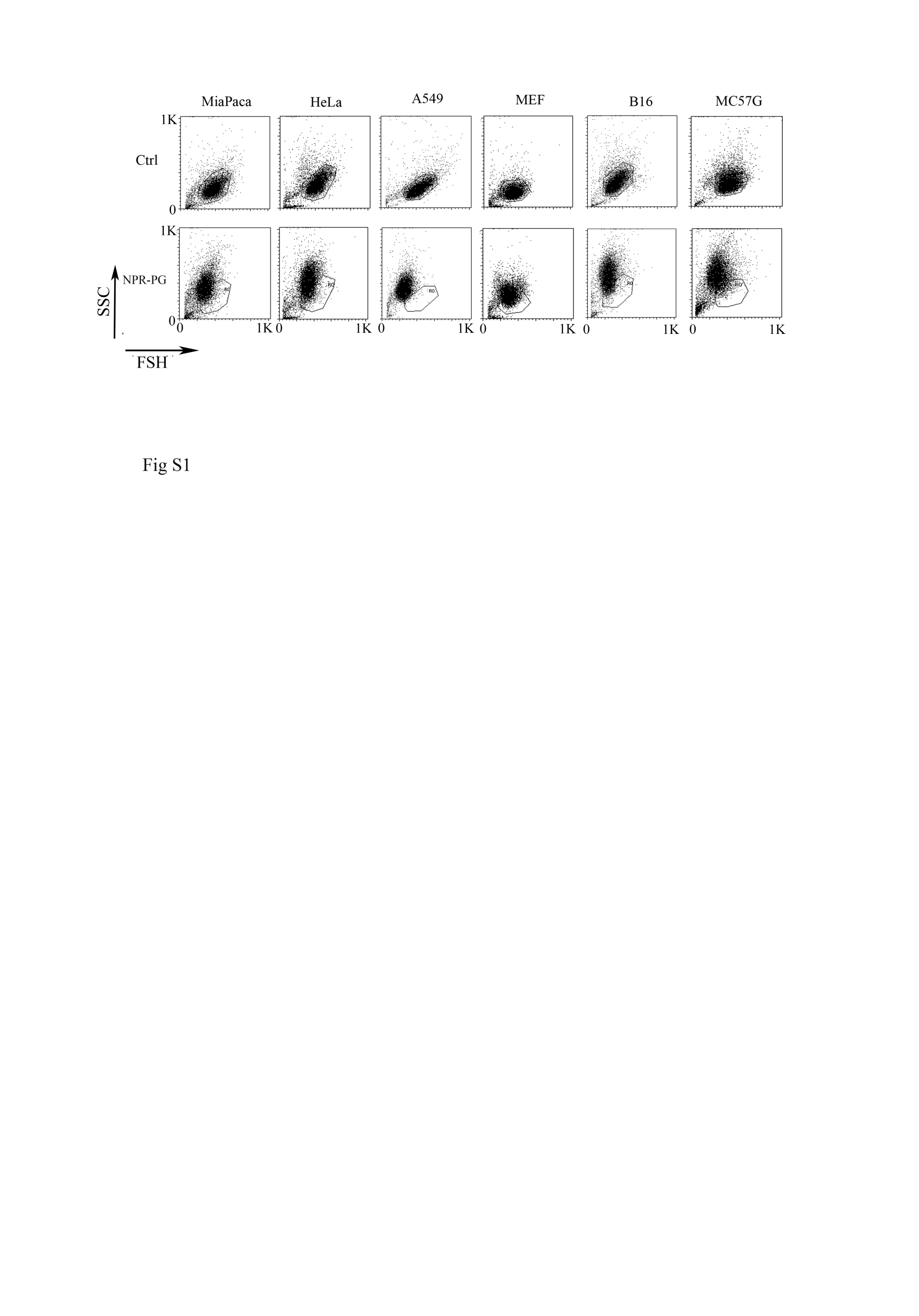


Figure S5. (A) Analysis of cell morphology changes by flow cytometry. MiaPaca, HeLa, A549, MEF, B16, MC57G line cells were incubated with four types of nanoparticles (NPR-P, NPR-PG, NPR-PT, NPR-PTG) at four concentrations (25, 50, 100 and 200 µg/mL) for 48 hours as indicated in experimental section. After incubation time, cells were analyzed by flow cytometry. A representative experiment is shown at the concentration of 200 µg/mL *Note:*  Ctrl = negative control.

**3: Glucogen deposit.**

The presence of glucogen deposits in the vacuoles observed in the livers of mice treated with NPRs was discarded employing a glucogen specific dye, periodic acid-Schiff (PAS) in combination with diastase (PAS/D), an enzyme that breaks down glycogen Fig S6.

Figure S6: Periodic acid-Schiff (PAS) and periodic acid-Schiff diastasa (PAS/D) stain of the liver of mice treated with NPRs. Mice were injected (i.v) with 6 µg/g NPR-PTG and sacrificed after 4 months, the organs were fixed and processed for PAS staining, as indicated in experimental section. Representative images are shown.

1. Pelaz B, Grazu V, Ibarra A, Magen C, del Pino P, de la Fuente JM: **Tailoring the synthesis and heating ability of gold nanoprisms for bioapplications.** *Langmuir* 2012, **28:**8965-8970.

2. Perez-Hernandez M, Del Pino P, Mitchell SG, Moros M, Stepien G, Pelaz B, Parak WJ, Galvez EM, Pardo J, de la Fuente JM: **Dissecting the molecular mechanism of apoptosis during photothermal therapy using gold nanoprisms.** *ACS Nano* 2015, **9:**52-61.
